# Are stronger memories forgotten more slowly? No evidence that memory strength influences the rate of forgetting

**DOI:** 10.1371/journal.pone.0200292

**Published:** 2018-07-13

**Authors:** Haggar Cohen-Dallal, Isaac Fradkin, Yoni Pertzov

**Affiliations:** Department of Psychology, The Hebrew University of Jerusalem, Jerusalem, Israel; Coventry University, UNITED KINGDOM

## Abstract

Information stored in visual short-term memory is used ubiquitously in daily life; however, it is forgotten rapidly within seconds. When more items are to be remembered, they are forgotten faster, potentially suggesting that stronger memories are forgotten less rapidly. Here we tested this prediction with three experiments that assessed the influence of memory strength on the rate of forgetting of visual information without manipulating the number of items. Forgetting rate was assessed by comparing the accuracy of reports in a delayed-estimation task following relatively short and long retention intervals. In the first experiment, we compared the forgetting rate of items that were directly fixated, to items that were not. In Experiments 2 and 3 we manipulated memory strength by extending the exposure time of one item in the memory array. As expected, direct fixation and longer exposure led to better accuracy of reports, reflecting stronger memory. However, in all three experiments, we did not find evidence that increased memory strength moderated the forgetting rate.

## Introduction

Visual Short-Term Memory (STM) refers to temporal retention of visual information no longer present in the environment [1] and is a crucial ability in many everyday tasks. However, the amount of information that can be maintained in visual STM is very limited. Most research has assessed memory performance following a fixed retention interval and explored memory limitations in terms of the number of objects [2–4] or the amount of information [5–7] that can be maintained. However, in the past few years, a growing number of studies have addressed the temporal limitations of visual STM as captured by the loss of information within the span of a few seconds(i.e., rapid forgetting; [8–12]).

Studies have shown that the rate at which information is forgotten varies across individuals [13–15], is reliable and stable across different testing sessions [16] and accounts for differences in working memory span [17]. Most studies have assessed the rate of forgetting by measuring reports after different retention intervals. When the different retention intervals are interleaved within the same block of trials, all processes that occur before or after the maintenance stage are equivalent in all conditions. Therefore, any increase in reporting errors following a relatively longer retention interval can only be attributed to the extended interval (i.e. forgetting) and not to other processes related to encoding or retrieval.

A recent study showed that memory load strongly influences the rate of forgetting [9]. A single item was shown to be maintained in memory with a minor decrease in report accuracy over short time spans; however, the addition of items to memory enhanced time-dependent degradation. This may occur due to competition between items that reside together in memory, or alternatively because of lower memory strength of each item due to competition in encoding into memory [9]. The current study was designed to distinguish between these two alternatives by manipulating the initial memory strength of an item while keeping the number of items to be remembered fixed. An effect of memory strength on the rate of forgetting will imply that the enhanced forgetting may indeed be a result of the initial degradation of memory following competition at encoding. However, if the rate of forgetting is not affected by initial memory strength this may imply that the effect of additional items on the rate of forgetting is not due to their initial memory strength but rather due to increased competition over maintenance resources.

Not all studies have reported rapid forgetting (e.g., [18]). Forgetting has primarily been found when information cannot be easily rehearsed and maintained [19–21]. For example, Ricker and Cowan [22] reported rapid forgetting when unconventional letters were memorized (i.e. non- English letters for English speakers), but not in the case of English letters. Presumably, this is because unconventional letters lead to less efficient maintenance strategies than conventional, familiar letters.

Forgetting is also typically observed in delayed estimation tasks (e.g., [12,23–25])in which a visual array is shown (e.g., patches of color or line bars in different orientations) and after a blank retention interval, participants are required to report a feature of a specific stimulus on a continuum (e.g., colored circles or bar orientations, respectively). This task enables researchers to extract much more information than from a binary answer, provided by traditional visual short term memory tasks (e.g. the "change detection" task) in which participants indicate whether a change between two consecutive visual arrays was detected [26,27]. The distribution of report errors around the correct values has been used in order to quantify the quality of memory, with narrower distributions reflecting more precise memory reports. Bays and collegues [28] found that the amount of time items are displayed influences their precision of recall. Regardless of the number of items to be remembered, precision of recall was lower when the exposure duration of an item was shorter than 300 ms, implying an encoding limitation. Hence, memory strength can be manipulated by modulating exposure duration, in such a manner that different levels of display durations can lead to different levels of memory strength. Unfortunately, in Bays and collegues [28], reports were only measured after a single retention interval, precluding any assessment of forgetting rate. In the current study we will use the effect of display duration on precision in order to modulate memory strength.

Only a handful of studies have combined the three methodological pillars of (1) unconventional visual stimuli, (2) variable retention intervals, and (3) a delayed estimation tasks [8,9,12,29]. Indeed, these have led to several novel insights in the field of rapid visual forgetting. A pioneering study by Zhang and Luck[12] used a memory array of three colored squares with variable retention intervals and reported significant time-based forgetting of visual items. However, their main focus was to distinguish between two alternative processes that might occur during forgetting: gradual decay of information vs a complete loss of the information. By demonstrating that most of the additional errors at extended delays could be attributed to random guessing they claimed that information is completely lost from memory. Unfortunately, they did not address the putative factors influencing the rate of forgetting.

Souza and Oberauer [29] focused on another debate regarding the factors involved in forgetting and investigated if forgetting is caused by the mere passage of time(e.g., [22,30–33]), or is it due to interference (e.g., [34–37]). They used a similar delayed estimation task to Zhang and Luck [12] but here, apart from the manipulation of the retention interval they also manipulated the inter-trial-interval. Doing so, they showed that not only the length of the retention interval affects rapid forgetting but also the spacing between trials that relates to the temporal distinctiveness between items on the psychological timeline. This conclusion has since been contradicted [38], and the debate continues.

**Fig 1:**
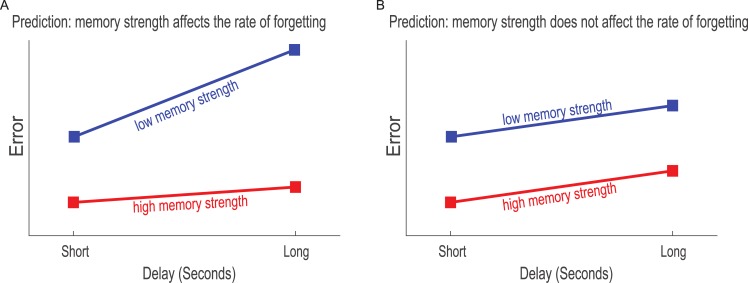
Predictions. The two hypothesized outcomes: (A) If stronger memories are forgotten more slowly, the rate of forgetting (as measured by angular error) should be steeper in the low memory strength condition than in the high memory strength condition. (B) If stronger memories are not forgotten more slowly the forgetting rate (as measured by angular error) should be comparable across memory strength conditions.

Critically, none of these studies tested whether the strength of a memory representation influences the rate of rapid forgetting. This question is inherent for understanding the processes behind forgetting and answering it might provide critical constrains on the different models suggested for forgetting. Here we conducted three delayed estimation experiments with variable retention intervals and compared the forgetting rate in two conditions that were expected to lead to two different levels of memory strength, as reflected in the recall error of the reports. High and low memory strength were operationally defined as conditions with lower and higher recall error, respectively. Specifically, we tested whether differences in memory strength change the rate of forgetting, which was defined as the difference in recall error between long and short retention intervals. We posited that if stronger memories are forgotten more slowly, there should be shallower forgetting slopes when memory strength is high (see Fig 1A for an illustration). Similarly, if the forgetting rate is not affected by memory strength the slopes should be comparable when memory strength is high and low (Fig 1B).

In Experiment 1 the trials were divided into two memory strength conditions defined by whether an item was or was not directly fixated during the display of the memory array [39], whereas in Experiments 2 and 3 memory strength was manipulated by changing the display time of the target [40]. We focused on the interaction between the effect of retention interval and the strength of memory. An interaction would suggest that forgetting rate is dependent upon memory strength (See Fig 1A). This result would undermine the hypothesis that forgetting may be related to competition during memory and would suggest that forgetting can be related to competition during encoding. On the other hand, the absence of an interaction would suggest that forgetting rate, when measured by participants’ angular deviations, is not modulated by memory strength (see Fig 1B). This would be in line with the hypothesis that forgetting is caused by competition between items that reside together in memory [9]. Since differentiating the two hypotheses may involve evidence supporting the absence of an interaction, frequentist inference is insufficient. Therefore, we used Bayesian inference to establish the credibility of an interaction in light of the data. Bayesian inference is also highly suitable for aggregating evidence across several experiments, which provides more confident estimates of the parameters of interest. Thus, Bayesian inference was conducted to analyze both the results of individual experiments and the evidence that aggregated across experiments.

## Experiment 1

Research has shown that the more participants fixate an item, the better their accuracy of recall (e.g., [39]). In this experiment, we used eye tracking to monitor fixations, and predicted that items that were directly fixated would be remembered better than items that were not fixated. Recall error was measured after two different retention intervals. We hypothesized that error rates would be lower after short retention intervals than long retention intervals, showing forgetting over time. To explore the main question of the current study, we used frequentist and Bayesian statistical approaches to examine whether the forgetting slopes were similar or different in the two memory strength conditions.

### Method

#### Participants

Twenty-two neurologically normal participants (age range 18–28 years, mean 22.6 ± 2.9) participated in the experiment after providing written informed consent. This study was approved by the ethics committee of the Psychology Department at the Hebrew University of Jerusalem, Israel. All participants reported normal or corrected-to-normal visual acuity and had normal color vision (assessed using the Ishihara 1936 test for color deficiencies). All were students at the Hebrew University and were paid 40 NIS (approximately $10.00) for one hour of their time.

#### Materials

The stimuli were displayed using Matlab and the Cogent toolbox (Wellcome Department of Imaging Neuroscience, London, UK) on a 23'' Syncmaster monitor, with a 120 Hz refresh rate, and a 1280 X 1024 screen resolution. The monocular gaze position was tracked at 1000 Hz with an Eyelink eye tracker 1000 plus (SR Research Ltd., Mississauga, Ontario, Canada). Subjects sat at a viewing distance of approximately 70 cm with their head positioned on a chin rest.

#### Procedure

**Fig 2:**
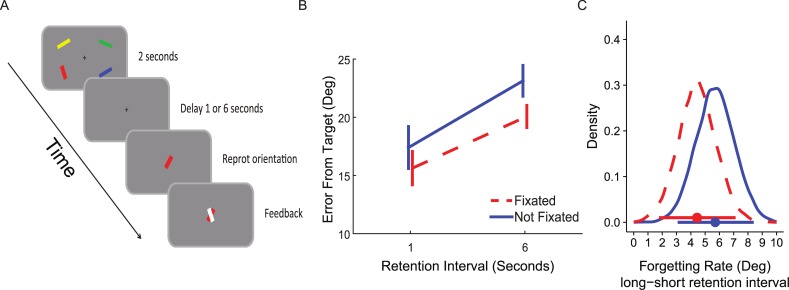
Method and results of Experiment 1. (A) Sample trial. A memory array was followed by a blank retention interval with variable durations. When a probe appeared, participants were required to rotate it to match the orientation of the target bar. (B) Average error (and SEM across participants) for each memory strength condition and retention interval condition. (C) The posterior distributions of the simple effects of retention interval (long–short) for the two memory strength conditions. Horizontal bars represent the 95% high posterior density intervals (HDI). The overlap in the two distributions supports the notion that there is no difference in forgetting rate between the conditions.

The experimental design is presented in Fig 2A. Each trial began with a central black fixation cross (0.4° diameter) displayed for 1000 ms over a gray background. Then a memory array appeared for 2000 ms consisting of four oriented bars (0.4° x 2.0°) in 4 different colors (blue, red, yellow, black) that were randomly distributed on an imaginary circle (radius: 8.2°) around the fixation cross, at equal distances from the center of the bars. The orientation of each bar was independently and randomly chosen from a circular parameter space of 0–180° (i.e., the full range of possible undirected bar orientations) at a one degree resolution. Next, a blank retention interval was introduced for 1000 or 6000 ms (retention intervals were randomly assigned to each trial), followed by a single bar (probe) presented in the center of the screen in a random orientation in one of the four colors of the bars in the memory array. Participants were required to rotate the probe using the mouse to match it to the remembered orientation of the bar with the probe color (i.e. the target bar). The target bar was randomly selected out of the four bars in the memory array. Once the participants were satisfied with the probe orientation, they pressed the space bar to proceed to the next trial. After their response, a white bar with the correct orientation appeared superimposed on the probe for 600 ms as feedback. Participants were instructed to be as accurate as possible regardless of the time it took them to adjust the probe orientation.

The experiment was divided into blocks of 60 trials. At the beginning of each block, the eye tracker was calibrated and then validated using the nine-point procedure provided by SR research (SR Research Ltd., Mississauga, Ontario, Canada). Calibration and camera adjustments were repeated if the average error during the validation process exceeded 1°. At the end of each block, feedback was displayed indicating the average accuracy of the participant’s reports on that block (the average angular error was linearly transformed to a 0–100 scale; 0 was the lowest accuracy and 100 was perfect adjustment). To encourage participants to engage in the task, they were told in advance that at the end of the experiment one of the experimental blocks would be randomly selected and if the score of that block was higher than 90%, they would receive a bonus of ten NIS (~$3.00). Each participant did 3–5 practice trials with the experimenter present in the room until it was clear that the participant understood the task. During the experiment, the experimenter sat in a control room and observed the display and eye tracker screens. Overall, each participant completed one hour of testing and a minimum of three blocks (180 trials). Direct fixations were defined as fixations within a circular interest area with a radius of 3.3° around the center of the target bar. The memory strength conditions were defined as whether the target item was directly fixated or not.

### Statistical analysis

Statistical analysis was conducted using both frequentist ANOVA and Bayesian inference. A two-factor repeated measure ANOVA was applied, with memory strength and retention interval as the within-subject factors. Bayesian inference was applied in two forms: Bayesian parameter estimation and Bayesian model comparison. In Bayesian parameter estimation, the current knowledge regarding parameters of interest is inputted, and the data are used to update these parameters [41–43]. In our case, the posterior distribution of the interaction coefficient was the major focus, specifically, the probability of a zero vs. non-zero interaction. We used reasonable non-informative priors for all parameters to prevent biasing of the posterior distribution by the prior of choice, such that it would only be determined by the results of the experiment. In addition, sensitivity analysis was conducted to verify the insensitivity of the posterior distributions to the choice of priors ([43,44]; see supporting information S1 Text).

In contrast to parameter estimation, Bayesian model comparison can be used to compare two hypotheses directly. Bayesian model comparison is usually conducted by computing a Bayes Factor (BF), which compares the relative probability of two models by computing the ratio of the marginal likelihoods of the data given both models. Marginal likelihood, which constitutes the denominator of the Bayes theorem, integrates the prior-weighted likelihood over the parameter space. Importantly, Bayesian model comparison is sensitive to the choice of priors, even when using non-informative priors. It is thus recommended to use reasonably informed priors for the parameters [44,45]. Therefore, we used both a non-informative prior (as is usually done in various BF analysis software), and a prior informed by a pilot study we conducted to examine the probable magnitude of an interaction effect (see supporting information S2 Text). We used JAGS [46] for both Bayesian parameters estimation and Bayesian model comparison. A detailed presentation of the statistical models used, as well as the different priors, can be found in the supporting information S3 Text.

### Results

We first defined two levels of memory strength by tracking the eye movements of the subjects during the memory array. We divided all the trials into two levels: trials in which the target item was fixated (mean of 66% of all trials; SD across participants = 19%), and trials in which the target item was not fixated (mean of 34% of the trials; SD = 19%). Thus, two levels of memory strength were defined by the presence or absence of a direct fixation on the target item.

Error was defined as the angular deviation between the reported orientation and the true orientation of the target bar in the memory array. Errors were grouped and averaged separately for each participant, memory strength condition and length of retention interval.

The repeated measures ANOVA with memory strength (high memory strength = direct fixation on the target, low memory strength = no fixation on the target) and retention interval (1000, 6000 ms) as within-participant factors (Fig 2B) showed a main effect for retention interval (*F*(1,21) = 27.6, *p* < .001, $\eta_{p}^{2}=.57$) reflecting substantial forgetting of information at extended intervals. We also found a main effect of fixations on the target (*F*(1,21) = 6.36, *p* = .02, $\eta_{p}^{2}=.23$) confirming that a direct fixation on an item increases accuracy of recall (i.e. memory strength). However, there was no significant interaction between retention interval and direct-fixation (*F*(1,21) = .57, *p* = .46, $\eta_{p}^{2}=.04$) suggesting that the different memory strength conditions exhibited similar forgetting rates.

**Fig 3:**
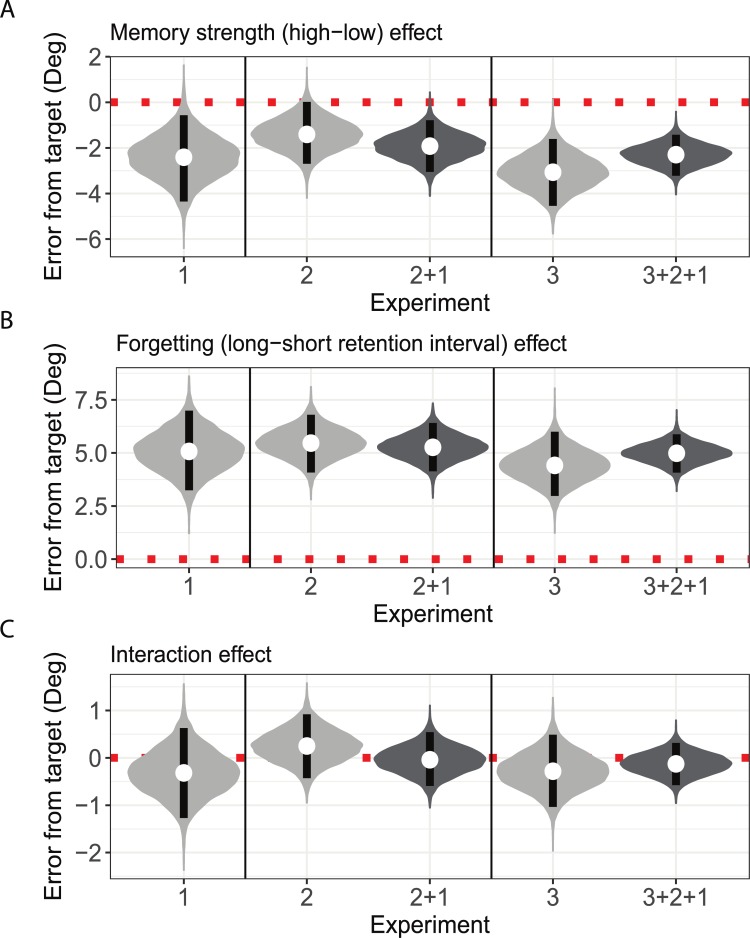
Bayesian parameter estimation and aggregation of evidence across experiments. Violin plots depicting the posterior distributions for the main effects of memory strength and retention interval as well as the two-way interaction. Light gray violins depict posterior distributions for the parameters given non-informative priors and data from the separate experiments. Dark gray violins depict posterior distributions for the parameters when aggregating evidence across experiments using the posterior of the previous experiments as the prior for the later experiments. White dots represent the center of the posterior distribution, and black rectangles depict 95% high posterior density intervals. The dashed red line marks the zero effect line. (A) Posterior distributions of the main effect of memory strength. (B) Posterior distributions of the main effect of retention interval (i.e. forgetting). (C) Posterior distributions for the retention interval and memory strength interaction. The distributions of the interaction effect overlap the zero effect line, supporting the notion that there was no difference in forgetting rate between the conditions.

As elaborated above, we used Bayesian parameter estimation to estimate the credible intervals for the main effects and interaction. As depicted in Fig 3A, the posterior credible difference between errors for the fixated and non-fixated targets was of -2.43° on average, with 95% high posterior density intervals (HDIs, which can be considered a Bayesian alternative to confidence intervals) of [-4.33°, -0.54°]. Hence, the results of Experiment 1 show that there is a 95% certainty that the credible difference between errors for fixated and non-fixated targets lies between -4.33° and -0.54°. The posterior credible difference between short and long retention intervals was 5.10° on average (95% HDI [3.29°, 6.96°]; see Fig 3B). The posterior distributions for the simple effects of the length of retention interval are depicted in Fig 2C. The forgetting rate was similar for both fixated (95% HDI [1.88°, 7.03°]) and non-fixated (95% HDI [3.08°, 8.29°]) targets. The posterior distribution of the interaction (Fig 3C) effect straddled zero, indicating a 95% certainty that the interaction lies between -1.19° and 0.63°, whereas the probability that the interaction is zero, or close to zero is relatively high.

Further support for the absence of an interaction effect was obtained by examining the BFs by comparing the model including an interaction coefficient to a model with no interaction term (i.e. interaction effect equals zero). When using a non-informative prior for the interaction effect the model with no interaction was 5.47 times more probable; when using an informed prior, the model with no interaction was 2.7 times more probable (see S3 Text for a specification of the priors).

### Discussion

In Experiment 1 memory strength was determined by the presence or absence of direct fixations on the target item during the memory array presentation. As expected, the presence of direct fixations had a significant effect on the error rate, such that the average error in the absence of a direct fixation was higher than for fixated targets. We also found the expected effect of retention interval duration, with a similar magnitude for both memory strength conditions. Thus, the absence of an interaction between the conditions (supported by both frequentist and Bayesian analyses) suggests that the level of memory strength did not affect the rate of forgetting. However, in Experiment 1 memory strength was defined post-hoc according to the participants’ scanning patterns. Thus, the split into two memory strength conditions was correlative in nature and hampered the assessment of a direct relationship between memory strength and forgetting rate. Experiment 2 was designed to better control the classification of trials into the two memory strength conditions.

## Experiment 2

The goal of Experiment 2 was to modulate memory strength using a planned manipulation, rather than investigating a post-hoc classification into memory strength conditions. In this experiment, one of the items in the memory array was displayed slightly before the other three items, and then remained on the screen until the whole memory array disappeared. Hence, this item benefitted from increased exposure duration. This manipulation was expected to lead to a shift of visual processing resources towards this item as well as to better encoding, and thus eventually to a stronger memory representation as reflected in lower recall errors [40]. Each one of the four items had 25% of being probed. When the probed item (target) was the item presented prior to the rest of the memory array; these trials were part of the high memory strength condition. All the other trials, in which the target item was one of the other three items in the memory array, were of the low memory strength condition. As before, our main research question was whether the rate of forgetting would be similar or different in the two memory strength conditions. We expected to find an effect of retention interval and an effect of exposure duration. An interaction between the two factors would suggest that the memory strength indeed influences the rate of forgetting. Otherwise, a lack of an interaction would further strengthen the results from Experiment 1 that the rate of forgetting is not necessarily affected by memory strength.

### Method

#### Participants

Twenty-one neurologically normal participants (age range 19–27 years, mean 23.19 ± 1.9) participated in the experiment after providing written informed consent. This study was approved by the ethics committee of the Psychology Department at the Hebrew University of Jerusalem. All participants did not participate in Experiment 1 and reported normal or corrected-to-normal visual acuity and had normal color vision (assessed using the Ishihara 1936 test for color deficiencies). All were students at the Hebrew University and were paid 40 NIS (approximately $10.00) for one hour of their time.

#### Materials

The stimuli were displayed using Matlab and the Cogent toolbox (Wellcome Department of Imaging Neuroscience, London, UK) on a 28.5 x 51.0 cm DELL touch screen computer (Inspiron one 2320) with a resolution of 1080 x 1920 at a viewing distance of approximately 40 cm (39.2° x 65.0°).

#### Procedure

**Fig 4:**
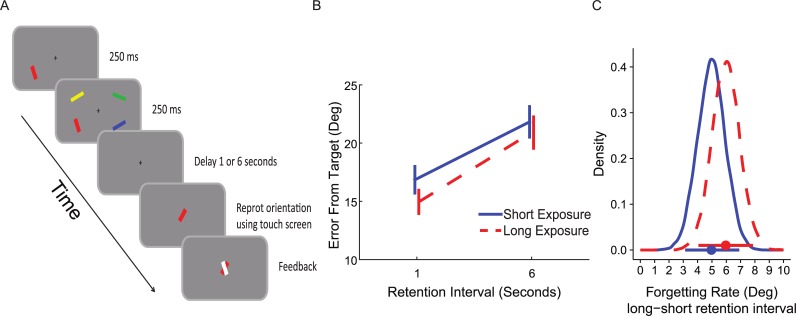
Method and results of Experiment 2. (A) Sample trial. One bar appeared before the rest of the memory array. After a blank retention interval in variable durations, a probe appeared and participants were required to rotate it to match the orientation of the target bar. (B) Average error (and SEM across participants) for each memory strength condition and retention interval condition. (C) The posterior distributions of the simple effects of retention interval (long–short) for the two memory strength conditions. Horizontal bars represent the 95% high posterior density intervals (HDI). The overlap in the two distributions supports the notion that there is no difference in forgetting rate between the conditions.

The experimental design is presented in Fig 4A. It was similar to the design in Experiment 1 except for the following changes: one randomly selected bar appeared 250 ms before the rest of the memory array, then the entire array consisting of the four oriented bars was presented for another 250 ms. The probed bar (target) was randomly selected from the four bars in the memory array. Therefore, the bar with the longer exposure duration was probed in 25% of the trials. Participants were instructed in advance that they would be asked about each one of the four bars randomly and that the appearance of one of the bars prior to the rest was irrelevant to the task. The experiment was divided into blocks of 60 trials; at the end of each block, feedback was displayed indicating the average accuracy on that block. All participants completed one hour of testing and a minimum of four blocks (240 trials).

### Statistical analysis

The statistical analysis was similar to that of Experiment 1, and was conducted using both frequentist ANOVA, and Bayesian inference. A two-factor repeated measure ANOVA was applied, with memory strength (high memory strength = longer exposure duration, low memory strength = shorter exposure duration) and retention interval (1000, 6000 ms) as within-subject factors. Bayesian parameter estimation was used for aggregating evidence across experiments, since the posterior distribution of one experiment can be used as the prior for a subsequent experiment. This procedure in most cases leads to a more stable and less uncertain conclusion regarding the parameters [43,47]. Thus, we report both the results of Bayesian inference for Experiment 2 alone, as well as the results obtained by aggregating the information derived from Experiments 1 and 2.

Studies of visual STM frequently use modeling of the distribution of errors (e.g., [6,48–50]) and used a parameter of the distribution to quantify the precision at which items were recalled. Modeling led to qualitatively similar results as the averaged error and therefore strengthen our interpretation, but do not shed new light on the theoretical question explored in this study. Therefore, the results based on modeling the responses are reported in the supporting information S4 Text.

### Results

A repeated measures ANOVA with memory strength and retention interval as within-participant conditions, revealed the expected main effects of retention interval (*F*(1,20) = 40.50, *p* < .001, $\eta_{p}^{2}=.67$) and memory strength (*F*(1,20) = 6.20, *p* = .02, $\eta_{p}^{2}=.24$). Thus, confirming that shorter retention intervals and longer exposure durations decrease error rates. As in Experiment 1, there was no significant interaction between retention interval and memory strength (*F*(1,20) = .92, *p* = .34, $\eta_{p}^{2}=.04$), indicating that the rate of forgetting was similar in the different memory strength conditions (see Fig 4B).

As in Experiment 1, we used Bayesian parameter estimation to estimate the credibility values of these effects. As depicted in Fig 3A, the posterior difference between trials with high vs. low memory strength was -1.42° on average (95% HDI [-2.76°, -0.13°]). The posterior difference between trials with a short vs. long retention interval was 5.48° on average (95% HDI [4.15°, 6.78°]; see Fig 3B). Thus, the data supported the assumption of both memory strength and retention interval effects. The posterior distribution of the interaction effect, as well as its HDI (95% HDI [-0.42°, 0.9°]; see Fig 3C) supported the absence of an interaction. Furthermore, as depicted in Fig 4C, the simple effects of retention interval were similar across memory strength conditions (High memory strength 95% HDI [4.12°, 7.76°]; Low memory strength 95% HDI [3.14°, 6.88°]. Interestingly, the pattern of results was different from what was found in Experiment 1, in that the retention interval had a slightly larger (and highly overlapping) effect in the high memory strength condition. This inconsistency in the direction of difference in the rate of forgetting reinforced the conclusion of the absence of a stable interaction effect. Further support for this conclusion emerged when examining the BF, which showed that the model with no interaction was 7.31 or 14.41 times more likely for non-informative and informative priors, respectively (see S3 Text for a specification of the priors). Note that the relatively high BF when using informative prior, results from the fact that in Experiment 2, forgetting was slightly more pronounced when memory was stronger, in contrast to what one would expect if stronger memories were forgotten more slowly (an expectation that was manifested in the informative prior we have used, see S3 Text).

Bayesian analysis is also highly suitable for aggregating the results of multiple experiments. When we used the posterior distributions of Experiment 1 as the priors for Experiment 2, the uncertainty regarding all three parameters decreased, further supporting the conclusions from each experiment separately. That is, both memory strength (95% HDI [-3.01°, -0.76°]), and retention interval (95% HDI [5.26°, 4.1°]) had a strong effect on accuracy. However, memory strength did not moderate the effect of retention interval, since the posterior distribution of the interaction was centered on -0.04° (95% HDI [-0.63°, 0.5°]), suggesting that aggregating evidence from both experiments increased the confidence in the absence of an interaction effect (see Fig 3). Calculating BF on the aggregated results of Experiments 1 and 2, showed that the model with no interaction is 11.66 times more likely when using a non-informative prior, and 10.67 times more likely when using an informative prior, thus providing strong support for the absence of an interaction.

### Discussion

In Experiment 2, memory strength was manipulated by altering the exposure duration of an item. As expected, exposure duration as well as the length of the retention interval had clear effects on recall error. However, as in Experiment 1, there was no interaction between exposure and retention interval, implying that although the error rate was affected by retention interval, this effect was not moderated by exposure duration. These results suggest that memory strength did not affect the rate of forgetting, at least not using the error measurements we have used (see general discussion). A similar and even stronger conclusion was reached when aggregating evidence across Experiments 1 and 2. However, one concern that might be raised is that the size of the memory strength effects was relatively small and a larger effect might reveal a significant interaction. Experiment 3 was designed to answer this concern.

## Experiment 3

In Experiments 1 and 2, we showed that the forgetting rate is not affected by memory strength. In Experiment 3, our goal was to replicate the findings of the previous experiments when increasing the effect of memory strength. To this end, we used a stronger temporal manipulation where we increased the amount of time that the target item appeared prior to the memory array and reduced the display duration of the other items in the memory array. The stronger manipulation was expected to lead to larger differences in recall errors between the two memory strength conditions. However, if the forgetting rate is indeed not directly affected by memory strength this manipulation should not lead to different forgetting rates in the two memory strength conditions.

### Method

#### Participants

Twenty-one neurologically normal participants (age range 19–27 years, mean 22.95 ± 2.03) participated in the experiment after providing written informed consent. This study was approved by the ethics committee of the Psychology Department at the Hebrew University of Jerusalem. All the participants that participated in Experiment 3 did not participate in the two previous experiments. All participants reported normal or corrected-to-normal visual acuity and had normal color vision (assessed using the Ishihara 1936 test for color deficiencies). All were students at the Hebrew University and were paid 40 NIS (approximately $10.00) for one hour of their time.

#### Materials, procedure and statistical analysis

**Fig 5:**
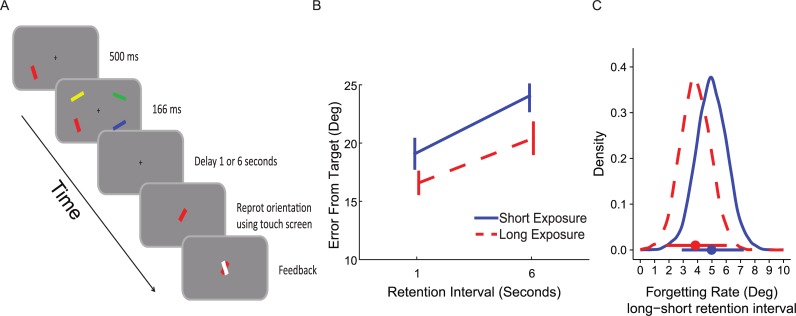
Method and results of Experiment 3. (A) Sample trial. One bar appeared before the rest of the memory array. After a blank retention interval in variable durations, a probe appeared and participants were required to rotate it to match the orientation of the target bar. (B) Average error (and SEM across participants) for each memory strength condition and retention interval condition. (C) The posterior distributions of the simple effects of retention interval (long–short) for the two memory strength conditions. Horizontal bars represent the 95% high posterior density intervals (HDI). The overlap in the two distributions supports the notion that there is no difference in forgetting rate between the conditions.

The materials and procedure used were identical to Experiment 2 except for one change: here one bar appeared 500 ms (instead of 250 ms) prior to the memory array, which appeared for 166 ms (instead of 250 ms). The procedure is illustrated in Fig 5A. The statistical analysis was also identical to Experiment 2, except for the inclusion of all three experiments in the aggregated Bayesian parameter estimation. This experimental design and analysis were preregistered in AsPredicted.org prior to data collection (pre-registration information is available at: https://aspredicted.org/ra8bb.pdf). As in experiment 2 modeling of the distribution of errors was performed and led to qualitatively similar results as the averaged error and are presented in S4 Text.

### Results

A repeated measures ANOVA revealed a main effect for retention interval (*F*(1,20) = 36.57, *p* < .001, $\eta_{p}^{2}=.65$) and exposure duration (*F*(1,20) = 11.34, *p* = .003, $\eta_{p}^{2}=.36$), reflecting fewer errors at longer exposure durations and shorter retention intervals. As in Experiments 1 and 2, there was no significant interaction between length of retention interval and memory strength (*F*(1,20) = 1.32, *p* = .26, $\eta_{p}^{2}=.06$), suggesting that the different exposure duration conditions did not alter the forgetting rate (see Fig 5B).

The posterior estimation of the difference between high and low memory strength trials using Bayesian parameter estimation was -3.07° on average (95% HDI [- 4.52°, -1.53°]; see Fig 3A). Note that this effect was twice as large as the corresponding effect in Experiment 2, reflecting the stronger temporal manipulations used in the current experiment exactly for this propose–to lead to a larger memory strength effect. The posterior credible difference between short and long retention interval was 4.4° on average (95% HDI [2.87°, 5.79°]; see Fig 3B). Finally, the posterior distribution of the interaction effect, as well as its HDI (95% HDI [-1.04°, 0.42°]) suggested, as before, that the probability of a near-zero interaction is high (see Fig 3C). Furthermore, as depicted in Fig 5C, the simple effects of the retention interval were similar for both memory strength conditions (High memory strength 95% HDI [3.51°, 6.01°]; Low memory strength 95% HDI [4.03°, 6.52°]. Finally, the BF comparison showed that a model with no interaction was 6.29 or 3.43 times more likely, for non-informative and informative priors, respectively.

The evidence across all three experiments confirmed these conclusions, and decreased the uncertainty regarding all three parameters even further (as depicted in Fig 3). There was 95% certainty that memory strength (averaged across the specific manipulation used) decreased errors between -3.18° and -1.42°, whereas extending the retention interval by 5 seconds increased errors to 4.07° and 5.85°. More importantly, the interaction was highly likely to be zero or close to zero (95% HDI [-0.58°, 0.30°]). Indeed, calculating BFs after aggregating the results of the three experiments resulted in a strong support for the absence of an interaction (a BF of 11.86 for non-informative priors, and a BF of 9.54 for informative priors).

### Discussion

The goal of Experiment 3 was to replicate the results of Experiment 2 with a stronger memory strength manipulation. As expected, the stronger modulation of exposure duration led to a larger difference in recall error between the two memory strength conditions. However, the larger effect did not lead to different rates of forgetting in the two conditions. Thus, in Experiment 3 we replicated the findings from Experiments 1 and 2, providing further support for the absence of memory strength modulation on the rate of forgetting.

## General discussion

The aim of the current study was to explore the relationship between the strength of an underlying memory representation and the rate of rapid forgetting. To this end, we conducted three experiments in which memory strength was operationalized in different ways. In the first experiment, memory strength was determined according to the presence or absence of a direct fixation on the target item. In the following two experiments, we manipulated memory strength by changing the exposure duration of the items within a trial. The most striking result was that the rate of forgetting, as measured by the increment of participants’ angular deviations, was similar in items with stronger vs. weaker memory strength. This result was supported by frequentist analysis, and more so, by Bayesian parameter estimation and Bayesian model comparison, which are better suited to providing support for the absence of an effect. Furthermore, aggregating the results across the three experiments strengthened the support in the lack of an interaction effect. Hence, in the three experiments we failed to find that the strength of the memory modulates the rate of forgetting. What might these results suggest about forgetting?

The lack of evidence for modulation constrains the type of process that might underpin the controversial mechanism of forgetting. The effect of memory load [9] and that of memory strength (as manipulated in the current study) seems to be strikingly different, as only the former was demonstrated to influence the rate of rapid forgetting. That is, using an experimental paradigm which is very similar to the current one (e.g. stimuli type, report procedure and measurement of error), Pertzov and colleagues [9]showed that attempting to memorize additional items in memory led to steeper forgetting rates. The authors argued that enhanced forgetting is due to increased competition over maintenance resources between items that reside simultaneously in memory. However, another possible explanation is that the faster rate of forgetting is not related to competition during maintenance but rather to the decreased level of encoding when additional items are to be remembered [28]. In other words, this alternative interpretation suggests that when more items are displayed in the memory array, the initial memory representation of each item is weaker and therefore it is forgotten faster. However, the current findings weaken this alternative explanation, because initial memory strength was not found to affect the rate of forgetting (using identical measures of error in both studies). Thus, it seems that memory load effects the rate of forgetting through a different process than memory strength. Implying that it is competition over maintenance processes rather than the initial memory strength that leads to enhanced forgetting when multiple items are memorized.

Thus, our results imply that the mechanism of forgetting relates to the competition between items that reside simultaneously in memory [9,51,52]. Moreover, the current results constrain the type of competition that can occur and discard the option of a simple omnibus competition between all items in memory, as this option implies that a stronger representation should be less affected by the other memory representations, and therefore forgotten more slowly. Our results demonstrate that items that had a stronger memory representation are forgotten at the same rate as other items. Therefore, other types of competition, that are not modulated by memory strength, should provide more compelling accounts.

Importantly, the failure to find an effect of memory strength on the rate of forgetting is compatible with findings from verbal short and long-term memory [53]. In the field of verbal long-term memory, Slamecka and McElree [54] asked participants to study a list of 56 words, while varying the number of study trials (one or three) and the retention interval (immediate, 1 and 5 days). They found strong effects for retention interval and number of study trials but no interaction. The authors concluded that the long-term forgetting of verbal lists is unaffected by their extent of learning. However, in a study of verbal short-term memory, Hellyer [55]manipulated the number of presentations (1, 2, 4, or 8) of consonants and the length of the retention intervals from 3 to 27 seconds. He measured the proportion of correctly recalled consonants and found that consecutive repetitions of a stimulus during encoding led to slower forgetting over time. These results initiated an intense debate. Bogartz [56] noted that the values that represent the length of the retention interval and accuracy should be monotonically scaled to fit the “psychological” value of time and accuracy. To help resolve this conundrum, he re-analyzed the Slamecka and McElree [54] results as well as the Hellyer [55] data and came to the conclusion that both datasets (Hellyer [55]in verbal short-term memory and Slamecka and McElree [54] in verbal long-term memory) supported the notion that the rate of forgetting did not depend on original learning.

This highlights the importance of the scaling function in interpreting the results of an interaction (or lack thereof). Indeed, Loftus [57] suggested that ordinal interactions, or the absences of them (as the results of the current study), cannot be interpreted as different scaling functions between psychological to measured variables may lead to seemingly opposite conclusions. Therefore, the results of the current study should be interpreted with caution, as other measurements of errors might lead to different conclusions. Having said that, the absence of interaction in the current study is especially informative when interpreted with regard to the previous study that found clear interaction between delay and memory load using similar experimental procedure and analysis [9].

Overall, these results suggest that the forgetting rate is not a direct function of memory strength; however, this does not mean that forgetting rate is fixed. Previous studies have shown that the forgetting rate can be modulated by top-down voluntary control. Pertzov and colleagues [8]showed that when relevant cues were presented after a memory array, the cued item was forgotten slower than other items that were not cued. Thus, while the forgetting rate does not seem to be modulated by memory strength, it is accessible to strategic control. A similar explanation evokes the role of attention in STM: the goal-relevant item might remain longer in the “focus of attention” (as defined by several theories [58–60]) and therefore be forgotten more slowly.

In Experiments 2 and 3, memory strength was manipulated by prolonging the display duration of a single item in the memory array. The increase in memory strength, as evidenced by the decreased recall error, could be a result of the increased time available for encoding or a result of attention deployment due to its distinctness among the other items in the array. Previous studies have shown that both attention cueing and encoding time improve the accuracy of report in similar tasks [6,28,61]. Nevertheless, the exact reason that led to the better recall of the item that was presented for a longer duration is not relevant to our main question. Regardless of the process that led to better recall of the selected item following short delays, this manipulation did not seem to alter the rate of forgetting.

In conclusion, in three different experiments we failed to find a direct influence of memory strength on the rate of forgetting. This suggests that the rate of forgetting is not tightly linked to the strength of the memory representation, or in lay terms: stronger memories are not necessarily more resistant to forgetting than weaker ones. This finding demonstrates that the increased rate of forgetting found in larger memory loads is not due to the decreased strength in which the items were encoded to memory, but rather due to competition between items in memory.

## Supporting information

S1 Text Sensitivity analysis. We conducted sensitivity analysis to verify that the choice of non-informative priors did not significantly alter the posterior distributions.
(PDF)

S2 Text A pilot experiment that quantifies the expected interaction between exposure duration and delay. This experiment was designed to generate an interaction between exposure duration and delay to be used as informed priors in the Bayesian inference. We used prior research that showed clear and robust modulation of forgetting rate and used it to make a small, but critical, change in the experimental paradigm of experiment 2.
(PDF)

S3 Text The Bayesian model. Bayesian linear regression.
(PDF)

S4 Text Model fit comparison. Many studies in the field have modeled the distribution of errors and used a parameter of the distribution to quantify the precision in which items were recalled. Here we compared three models: the variable-precision (VP) model, a 2 part mixture model and a 3 part mixture model.
(PDF)
