# Supplementary material for: Are stronger memories forgotten more slowly? No evidence that memory strength influences the rate of forgetting
Source: PLoS One. 2018 Jul 13;13(7):e0200292. doi: 10.1371/journal.pone.0200292 (PMC6044536; doi:10.1371/journal.pone.0200292)
Supplement: S1 Text — We conducted sensitivity analysis to verify that the choice of non-informative priors did not significantly alter the posterior distributions. (PDF) [file pone.0200292.s001.pdf]

S1 Text. Sensitivity analysis

We conducted sensitivity analysis to verify that the choice of non-informative priors did not significantly alter the posterior distributions. In addition to the original set of priors, we used three additional sets, by decreasing the variance of the priors for the fixed effects (Priors-2), and introducing uniform priors for the fixed (Priors-3) and random effects (Priors-4). Table A1 compares HDIs for the main effects and interaction across the four sets of priors. Evidently, the differences are negligible, demonstrating that in our case the choice of priors has a negligible effect on the posterior distributions.

Table A1.

Sensitivity analysis examining the effects of choosing different non-informative priors.

Exp.	Effect	Priors-1	Priors-2	Priors-3	Priors-4
		$\beta_1 \sim N(0, 100)$	$\beta_1 \sim N(0, 10)$	$\beta_1 \sim U(0,90)$	$\beta_1 \sim U(0, 90)$
		$\beta_2 \sim N(0, 100)$	$\beta_2 \sim N(0, 10)$	$\beta_2 \sim U(-90, 90)$	$\beta_2 \sim U(-90, 90)$
		$\beta_3 \sim N(0, 100)$	$\beta_3 \sim N(0, 10)$	$\beta_3 \sim U(-90, 90)$	$\beta_3 \sim U(-90, 90)$
		$\mu_{\beta 0} \sim N(0, 100)$	$\mu_{\beta 0} \sim N(0, 10)$	$\mu_{\beta 0} \sim U(-90, 90)$	$\mu_{\beta 0} \sim U(-90,90)$
		$\sigma^2_{res} \sim IG(0.001, 0.$	$\sigma^2_{res} \sim IG(0.001, 0.$	$\sigma^2_{res} \sim IG(0.001, 0.$	$\sigma_{res} \sim U(0, 90)$
		$\sigma^2_{\beta 0} \sim IG(0.001, 0.$ [illegible]	$\sigma^2_{\beta 0} \sim IG(0.001, 0.$ [illegible]	$\sigma^2_{\beta 0} \sim IG(0.001, 0.$ [illegible]	$\sigma_{\beta 0} \sim U(0, 90)$
1	M. strength (b1)	[-2.12 : -0.22]	[-2.1 : -0.23]	[-2.14 : -0.28]	[-2.17 : -0.3]
1	Delay (b2)	[1.58 : 3.48]	[1.56 : 3.41]	[1.63 : 3.49]	[1.62 : 3.48]

1	Interaction (b3)	[-1.27 : 0.61]	[-1.21 : 0.6]	[-1.24 : 0.59]	[-1.25 : 0.59]
2	M. strength (b1)	[-1.38 : -0.06]	[-1.33 : -0.02]	[-1.32 : -0.03]	[-1.38 : -0.04]
2	Delay (b2)	[2.02 : 3.38]	[2.02 : 3.36]	[2.07 : 3.4]	[2.08 : 3.4]
2	Interaction (b3)	[-0.4 : 0.91]	[-0.39 : 0.95]	[-0.39 : 0.91]	[-0.42 : 0.88]
3	M. strength (b1)	[-2.29 : -0.82]	[-2.24 : -0.78]	[-2.28 : -0.84]	[-2.25 : -0.78]
3	Delay (b2)	[1.47 : 2.96]	[1.44 : 2.93]	[1.49 : 2.95]	[1.4 : 2.9]
3	Interaction (b3)	[-1.07 : 0.42]	[-1.01 : 0.44]	[-1.08 : 0.43]	[-1.02 : 0.48]
