# Supplementary material for: Are stronger memories forgotten more slowly? No evidence that memory strength influences the rate of forgetting
Source: PLoS One. 2018 Jul 13;13(7):e0200292. doi: 10.1371/journal.pone.0200292 (PMC6044536; doi:10.1371/journal.pone.0200292)
Supplement: S2 Text — This experiment was designed to generate an interaction between exposure duration and delay to be used as informed priors in the Bayesian inference. We used prior research that showed clear and robust modulation of forgetting rate and used it to make a small, but critical, change in the experimental paradigm of experiment 2. (PDF) [file pone.0200292.s002.pdf]

S2 Text. A pilot experiment that quantifies the expected interaction between exposure duration and delay

This experiment was designed to generate an interaction between exposure duration and delay to be used as informed priors in the Bayesian inference. We used prior research that showed clear and robust modulation of forgetting rate and used it to make a small, but critical, change in the experimental paradigm of experiment 2. Pertzov and colleagues (2013) found that the goal relevance of an item can modulate the rate of forgetting. They showed that a cue signaling the probable item to be probed changes the rate of forgetting; specifically, the most probable item to be cued was forgotten slower than the other items in the memory array. Here, we manipulated the relevance of the bar with the longer exposure duration in a similar manner, thus leading to different forgetting slopes in the two memory strength conditions. In Experiment 2 the exposure duration of the item was irrelevant to the task and participants were asked about the item with longer exposure duration at the same frequency as they were asked about any other bar in the memory array (25% of the trials). In the pilot experiment, we used the same design as in Experiment 2 except that the item with a longer exposure duration was probed in the majority of the trials (85% of the trials). Moreover, subjects were briefed before the experiment that they would be asked more frequently about the item that appeared first. Thus, the exposure duration in Experiment 2 also served as a "relevance cue". This was expected to lead to an interaction between exposure duration and delay, as the item that was displayed longer was also more relevant and forgotten more slowly. This interaction was later used as an informed prior in the Bayesian inference that studied the strength of evidence for such interaction.

Method

Twenty neurologically normal participants (age range years 18-25, mean 22.25 ± 1.67) participated in this experiment after providing informed consent. All participants reported normal or corrected-to-normal visual acuity and had normal color vision (assessed using the Ishihara 1936 test for color deficiencies). All were students at the Hebrew University and were paid 40 NIS (approximately $10.00) for one hour for their time.

The experimental design and statistical analysis were exactly the same as in Experiment 2, apart from a small change: in this experiment, the longer exposure condition consisted of 85% of the trials. Thus, out of the four bars that appeared in every trial there was an 85% chance that the probed bar would be the bar that appeared first, and each one of the other three bars only had a 5% chance of being probed .

Results

We replicated the analysis procedure used in Experiment 1, 2 and 3. First, we calculated the mean absolute error in each condition, and applied a repeated measures ANOVA with exposure condition (long, short) and delay (1000, 6000 ms) as within-participants factors (figure 3A). The two main effects were significant, delay: $F(1,19) = 83.9$, $p < .001$, $\eta_p^2 = .82$; exposure time: $F(1,19) = 52.81$, $p < .001$, $\eta_p^2 = .74$. However, unlike in Experiment 1, 2, and 3 the interaction between delay and exposure time was also significant, $F(1,19) = 12$, $p = .003$, $\eta_p^2 = .389$, implying that the manipulation succeeded in generating different rates of forgetting in the two exposure conditions.

We performed Bayesian parameter estimation to get posterior distributions for the interaction effect, that were later used as an informed prior in the BF analysis of the main

experiments. This resulted in a posterior distribution for the interaction coefficient with a mean of -1.25° and a variance of 0.396. These values were used to inform the interaction prior distribution in the BF analysis.

The posterior credible difference between short and long delay was 6.05 on average (95% HDI [3.68°, 8.43°]), and the posterior credible difference between the short and the long exposure conditions was 13.16° on average (95% HDI [10.81°, 15.59°]). Note that the effect of exposure duration was nearly 5 times larger than the respective effects in experiments 1-3, reflecting the fact that in this pilot study, exposure duration manipulated not only memory strength but mostly served as a relevance cue.

Discussion

This experiment replicates the finding that forgetting rate can be modulated by goal relevance. In this experiment, items that were presented for a longer duration and were more frequently probed were forgotten slower than the items that were presented for a shorter duration and were less frequently probed. The length of delay and length of exposure duration affected memory accuracy and interacted with each other. This interaction was then used as informed priors in the Bayesian analysis of the experiments in the main text. Note that the analysis revealed that only in this experiment, but not in Experiments 1-3, did the two factors interact. Thus, only when it is beneficial to maintain one item (it is more likely to be probed) it is forgotten more slowly.

References

1. Pertzov Y, Bays PM, Joseph S, Husain M. Rapid forgetting prevented by retrospective attention cues. J Exp Psychol Hum Percept Perform. 2013;39: 1224–1231. doi:10.1037/a0030947
