# Supplementary material for: Are stronger memories forgotten more slowly? No evidence that memory strength influences the rate of forgetting
Source: PLoS One. 2018 Jul 13;13(7):e0200292. doi: 10.1371/journal.pone.0200292 (PMC6044536; doi:10.1371/journal.pone.0200292)
Supplement: S3 Text — Bayesian linear regression. (PDF) [file pone.0200292.s003.pdf]

S3 Text. The Bayesian model

We used Bayesian linear regression, after contrast coding delay (x1) and memory strength (x2). To account for the within-subject design, intercepts were allowed to vary between participants, therefore each participant's error is derived by:

$$Y_{ij} = \beta_{0_j} + \beta_1 * x1 + \beta_2 * x2 + \beta_3 * x1 * x2 + \epsilon_{ij}$$

$$\beta_{0_j} \sim N(\mu_{\beta 0}, \sigma^2_{\beta 0})$$

$$\epsilon_{ij} \sim N(0, \sigma^2_{res})$$

Where Y_{ij} is the error size of the jth participant in the ith condition, $\mu_{\beta 0}$ is the overall mean error size, $\sigma^2_{\beta 0}$ is the between-subject variance in mean error size, and σ^2_{res} is the residual variance. Non-informative priors were used for all parameters, for this reason all regression coefficients (fixed effects) were assumed to have a normal prior distribution, with high uncertainty (i.e. $N(0{,}100)$), where ~100% of the distribution lies between -90 and 90, to reflect the natural bounds of the scale of absolute error. Variances (random intercept and error variance) were assumed to have a non-informative inverse gamma prior distribution (i.e. IG (0.001,0.001)). Since in contrast coding the coefficient represents half of the difference between groups, all reported simple effects were the coefficients multiplied by 2.

The full model code is as follows:

```
model{
for (i in 1:nTrials){
        Error[i]~dnorm(mu[i],resTau)
```

```
    mu[i]<-b0[subject[i]]+b1*Encoding[i]+b2*Delay[i]+b3*Encoding[i]*Delay[i]
}
for (s in 1:nSubj){b0[s]~dnorm(B0,b0Tau)}

B0~dnorm(0,0.01)T(0,)
b1~dnorm(0,0.01)
b2~dnorm(0,0.01)
b3~dnorm(0,0.01)
b0Tau~dgamma(0.001,0.001)
resTau~dgamma(0.001,0.001)
}
```

Bayes factors were computed in JAGS by introducing a categorical parameter M that corresponds to the relative probability of the two compared models, so that M1/M0 gives the Bayes factor [1]. Analysis was conducted in a nested manner; each model was compared to a simpler model (i.e. H0), in which b3 (the interaction) was constrained to zero. Bayes factors were computed by using both a non-informative prior on the interaction, that is $b3 \sim N(0,10)$, and an informative prior. Note that we used a less non-informative prior than in the parameter estimation, since using too non-informative priors inflates the Bayes factors. A prior with a variance of 10 is realistically non-informative since given a standard deviation that ranged between 6.43 and 7.33 across experiments, it implies an expectation of 67% that the effect size will be of medium size (i.e. +/-3 = 0.5*SD). The informative prior was derived from the posterior of b3 in the pilot study described above. The prior used was thus b3 ~ N(-1.251,

0.396) This prior represents the interaction effect one should expect under conditions that result such an interaction.

The full model code is as follows (note that b3prior was adjusted as described above):

```
model{
for (i in 1:nTrials){
	Error[i]~dnorm(mu[i,m],resTau)
	mu[i,2]<-b0[subject[i]]+b1*Encoding[i]+b2*Delay[i]+b3*Encoding[i]*Delay[i]
	mu[i,1]<-b0[subject[i]]+b1*Encoding[i]+b2*Delay[i]
}

for (s in 1:nSubj){b0[s]~dnorm(B0,b0Tau)}
B0~dnorm(0,0.1)
b1~dnorm(0,0.1)
b2~dnorm(0,0.1)
b3~dnorm(0,0.1)
m~dcat(mPriorProb[])
mPriorProb[1]<-0.5
mPriorProb[2]<-0.5
b0Tau~dgamma(0.001,0.001)
resTau~dgamma(0.001,0.001)
```

The models were run with 3 chains, included a burn-in of 1000 samples, and 10,000 retained samples with a thinning of 3 samples (meaning that only every 3rd sample is retained, to reduce memory usage). Convergence was tested by visually examining chains' mixing, as well

as by inspecting the Gelman-Rubin statistic, for which all point estimates were equal or smaller than 1.01, and lower than their respective upper CIs (representing the null hypothesis that the chains converge).

References

1. Kruschke JK. Doing Bayesian data analysis: A tutorial with R, JAGS, and Stan. Academic Press; 2014.
