# Supplementary material for: Are stronger memories forgotten more slowly? No evidence that memory strength influences the rate of forgetting
Source: PLoS One. 2018 Jul 13;13(7):e0200292. doi: 10.1371/journal.pone.0200292 (PMC6044536; doi:10.1371/journal.pone.0200292)
Supplement: S4 Text — Many studies in the field have modeled the distribution of errors and used a parameter of the distribution to quantify the precision in which items were recalled. Here we compared three models: the variable-precision (VP) model, a 2 part mixture model and a 3 part mixture model. (PDF) [file pone.0200292.s004.pdf]

S4 Text. Model fit Comparison

Many studies in the field have modeled the distribution of errors and used a parameter of the distribution to quantify the precision in which items were recalled. Here we compared three models: the variable-precision (VP) model [1], a 2 part mixture model [2] and a 3 part mixture model [3]. The variable precision model was fit using code taken from: http://www.ronaldvandenberg.org/code.html; [4,5]. The mixture models were fit using the MemToolbox ([6]; memtoolbox.org). The models were compared using two penalty based comparison measures: the Akaike Information criterion (AIC; [7]) and the Bayesian information criterion (BIC; [8]). In such comparisons, the model that yields the smallest scores is considered to provide the best account of the data. Both measures were extracted for each subject in each of the four conditions (two exposure conditions and two delay conditions). Then the final score for each subject was calculated as the sum of the measure (either the AIC or BIC measures) of the four conditions. The results are presented in Table A2 and Table A3. The results of the comparison using the AIC and the BIC were consistent: for all 42 subjects in Experiments 2 and 3 the VP model yielded the best fit (1 subject's model fit converged only using the VP model) and thus this model was further used in the analysis.

In Experiment 1 we did not perform any model fitting because of the low number of trials in the no fixation conditions (in the short and long retention intervals separately). In this experiment, the overall number of trails was lower than in the other experiments (Mean = 192.36, SD =17.42) because the calibration procedure between each block required time. Furthermore, because the separation between the conditions was done post hoc (if the bars were fixated or not), in most subjects (21 out of 22) the number of trials in the no fixation

conditions was 40 or less, which is not sufficient for model fitting. All these problems were eliminated in experiments 2 and 3.

Table A2

Model comparison between the three models using the AIC measure

Subject	Experiment	2 Part Mixture Model	3 Part Mixture Model	variable-precision (VP) model	Best Model Fit
1	2	3310.20	3313.13	527.58	VP Model
2	2	2520.51	2528.51	114.66	VP Model
3	2	2668.00	2672.49	212.16	VP Model
4	2	2541.33	2547.30	411.32	VP Model
5	2	3270.16	3277.28	470.71	VP Model
6	2	3065.71	3073.71	229.37	VP Model
7	2	3191.34	3199.10	479.57	VP Model
8	2	3192.65	3200.08	421.36	VP Model
9	2	2445.18	2453.04	184.64	VP Model
10	2	2485.89	2492.20	221.52	VP Model
11	2	2530.67	2538.62	281.28	VP Model
12	2	2302.24	2308.65	34.03	VP Model
13	2	2727.37	2734.25	485.54	VP Model
14	2	2790.30	2798.34	-49.11	VP Model
15	2	2980.30	2986.68	337.47	VP Model
16	2	3188.75	3195.54	377.16	VP Model
17	2	3238.87	3246.84	405.81	VP Model

18	2	3042.79	3049.57	208.63	VP Model
19	2	2879.59	2887.50	50.67	VP Model
20	2	3274.66	3279.12	450.98	VP Model
21	2	2701.46	2709.06	452.82	VP Model
1	3	2540.46	2543.23	7.22	VP Model
2	3	2723.01	2727.06	289.5	VP Model
3	3	3184.81	3179.59	503.63	VP Model
4	3	3388.22	3388.74	588.94	VP Model
5	3	3231.36	3233.67	411.42	VP Model
6	3	3213.46	3221.46	395.87	VP Model
7	3	3318.93	3326.35	504.08	VP Model
8	3	2732.6	2738.23	217.42	VP Model
9	3	3301.66	3309.66	486.31	VP Model
10	3	3446.51	3453.8	653.86	VP Model
11	3	3194.02	3202.02	363.11	VP Model
12	3	3382.36	3390.07	574.93	VP Model
13	3	3381.76	3384.89	576.04	VP Model
14	3	3267.71	3274.58	447.51	VP Model
15	3	3162.06	3166.52	334.28	VP Model
16	3	2892.97	2900.23	59.9	VP Model
17	3	2900.57	2908.39	107.85	VP Model
18	3	3281.4	3279.25	470.37	VP Model
19	3	3150.87	3156.03	343.13	VP Model
20	3	3181.56	3186.23	361.52	VP Model
21	3	3255.23	3259.6	452.41	VP Model

Note: AIC =3 Akaike's Information Criterion; NC = Model fit did not converge

Table A3

Model comparison between the three models using the BIC measure

Subject	Version	2 Part Mixture Model	3 Part Mixture Model	variable-precision (VP) model	Best Model Fit
1	2	3341.78	3360.50	552.89	VP Model
2	2	2551.11	2574.40	138.50	VP Model
3	2	2699.17	2719.25	236.86	VP Model
4	2	2571.05	2591.87	433.83	VP Model
5	2	3302.39	3325.61	497.00	VP Model
6	1	3097.86	3121.93	255.54	VP Model
7	1	3222.59	3245.98	504.39	VP Model
8	2	3224.29	3247.54	446.76	VP Model
9	2	2475.12	2497.96	207.50	VP Model
10	2	2516.27	2537.77	245.03	VP Model
11	2	2560.45	2583.29	303.89	VP Model
12	2	2332.77	2354.45	57.77	VP Model
13	2	2757.81	2779.90	509.14	VP Model
14	2	2822.07	2846.00	-23.51	VP Model
15	2	3011.88	3034.05	362.78	VP Model
16	2	3221.36	3244.45	404.02	VP Model
17	2	3271.15	3295.27	432.19	VP Model

18	2	3075.19	3098.16	235.16	VP Model
19	2	2911.43	2935.26	76.37	VP Model
20	2	3307.23	3327.96	477.77	VP Model
21	2	2732.05	2754.96	476.66	VP Model
1	3	2571.58	2589.92	31.85	VP Model
2	3	2753.78	2773.22	313.61	VP Model
3	3	3216.55	3227.2	529.18	VP Model
4	3	3419.64	3435.87	614.01	VP Model
5	3	3263.56	3281.98	437.67	VP Model
6	3	3245.41	3269.39	421.75	VP Model
7	3	3350.57	3373.8	529.48	VP Model
8	3	2763.67	2784.84	241.97	VP Model
9	3	3333.5	3357.43	512.02	VP Model
10	3	3478.95	3502.47	680.47	VP Model
11	3	3226.42	3250.62	389.66	VP Model
12	3	3414.34	3438.04	600.85	VP Model
13	3	3413.33	3432.25	601.34	VP Model
14	3	3299.99	3323.01	473.88	VP Model
15	3	3193.7	3213.98	359.68	VP Model
16	3	2925.35	2948.8	86.42	VP Model
17	3	2932.72	2956.61	134.01	VP Model
18	3	3313.64	3327.62	496.68	VP Model
19	3	3183.19	3204.5	369.55	VP Model
20	3	3213.87	3234.69	387.93	VP Model
21	3	3287.52	3308.03	478.79	VP Model

Note: BIC = Schwarz's Bayesian Information Criterion; NC= Model fit did not converge

Results

The distributions of errors were fit to the VP model and the parameter 1/J was extracted as a proxy for the breadth of the distribution of errors. We applied the same statistical analysis as on the absolute error; namely, a two-factor repeated measure ANOVA, with memory strength and retention interval as the within-subject factors. We also used Bayesian parameter estimation to examine the credible distribution of 1/J. See tables A4, A5 and A6 for the results. The VP model results replicate the results found with the average absolute error- a main effect of retention interval and a main effect of memory strength and no interaction between them. These results were found when using both ANOVA and Bayesian parameter estimation (in which only for the main effect the HDIs excluded zero).

Table A4

VP model Results of Experiment 2

**Effect**	**df**	**F**	**p**	**Effect size η_p^2**
Memory Strength	1, 20	5.06	0.035*	0.202
Delay	1, 20	11.86	0.002**	0.372
Memory Strength X Delay	1, 20	0.014	0.906	0.001

* $p < 0.05$, ** $p < 0.01$, *** $p < 0.001$

Table A5

VP model Results of Experiment 3

**Effect**	**df**	**F**	**p**	**Effect size η_p^2**
Memory Strength	1, 20	14.01	0.001**	0.412
Delay	1, 20	21.09	< 0.001***	0.513
Memory Strength X Delay	1, 20	0.01	0.915	0.001

* $p < 0.05$, ** $p < 0.01$, *** $p < 0.001$

Table A6

VP model Results – Bayesian parameter estimation

**Experiment**	**Effect**	**Posterior mean**	**95% HDI**
2	Memory Strength	-0.0109	[-0.0221, -0.003]
2	Delay	0.0221	[0.0112, 0.0334]
2	Memory Strength X Delay	0.0005	[-0.0103, 0.0114]
3	Memory Strength	-0.0273	[-0.0394, -0.0159]
3	Delay	0.0237	[0.0117, 0.0352]
3	Memory Strength X Delay	0.0005	[-0.0109, 0.0125]

References

1. van den Berg R, Shin H, Chou W-C, George R, Ma WJ. Variability in encoding precision accounts for visual short-term memory limitations. Proc Natl Acad Sci.

2012;109: 8780–8785. doi:10.1073/pnas.1117465109

2. Zhang W, Luck SJ. Discrete fixed-resolution representations in visual working memory. Nature. 2008;453: 233–5. doi:10.1038/nature06860
3. Bays PM, Catalao RFG, Husain M. The precision of visual working memory is set by allocation of a shared resource. J Vis. 2009;9: 7, 1–11. doi:10.1167/9.10.7
4. Berens P, Valesco MJ. The Circular Statistics Toolbox for Matlab. Tech Rep. 2009; 1–7. Available: http://www.kyb.tuebingen.mpg.de/fileadmin/user_upload/files/publications/attachments/MPIK-TR-184_[0].pdf
5. van den Berg R, Awh E, Ma WJ. Factorial comparison of working memory models. Psychol Rev. 2014;121: 124–149. doi:10.1037/a0035234
6. Suchow JW, Brady TF, Fougnie D, Alvarez G a. Modeling visual working memory with the MemToolbox. J Vis. 2013;13: 1–8. doi:10.1167/13.10.9.doi
7. Akaike H. A New Look at the Statistical Model Identification. IEEE Trans Automat Contr. 1974;19: 716–723. doi:10.1109/TAC.1974.1100705
8. Schwarz G. Estimating the Dimension of a Model. Ann Stat. 1978;6: 461–464. doi:10.1214/aos/1176344136
